# Polysaccharide Layer-by-Layer Coating for Polyimide-Based Neural Interfaces

**DOI:** 10.3390/mi13050692

**Published:** 2022-04-28

**Authors:** Eugenio Redolfi Riva, Angela D’Alessio, Silvestro Micera

**Affiliations:** 1 The BioRobotics Institute, Department of Excellence in Robotics and AI, Scuola Superiore Sant’Anna, 56127 Pisa, Italy; dalessioangela92@gmail.com (A.D.); silvestro.micera@santannapisa.it (S.M.); 2 Translational Neuroengineering, Centre for Neuroprosthetics and Institute of Bioengineering, School of Engineering, École Polytechnique Fédérale de Lausanne (EPFL), 1000 Lausanne, Switzerland

**Keywords:** nanostructured coating, layer-by-layer, neural interface, long-term biocompatibility, bioelectronic medicine, electrode–tissue interface

## Abstract

Implantable flexible neural interfaces (IfNIs) are capable of directly modulating signals of the central and peripheral nervous system by stimulating or recording the action potential. Despite outstanding results in acute experiments on animals and humans, their long-term biocompatibility is hampered by the effects of foreign body reactions that worsen electrical performance and cause tissue damage. We report on the fabrication of a polysaccharide nanostructured thin film as a coating of polyimide (PI)-based IfNIs. The layer-by-layer technique was used to coat the PI surface due to its versatility and ease of manufacturing. Two different LbL deposition techniques were tested and compared: dip coating and spin coating. Morphological and physiochemical characterization showed the presence of a very smooth and nanostructured thin film coating on the PI surface that remarkably enhanced surface hydrophilicity with respect to the bare PI surface for both the deposition techniques. However, spin coating offered more control over the fabrication properties, with the possibility to tune the coating’s physiochemical and morphological properties. Overall, the proposed coating strategies allowed the deposition of a biocompatible nanostructured film onto the PI surface and could represent a valid tool to enhance long-term IfNI biocompatibility by improving tissue/electrode integration.

## 1. Introduction

Neurological disorders and traumatic events to the nervous system represent a significant burden for the worldwide population and imply the loss or compromise of cognitive or sensory/motor functionality [1,2].

In the framework of the peripheral nervous system, a partial or total interruption of nerve continuity prevents the transmission of the action potential to the muscle fibers downstream of the injury, thus reducing the joint mobility of the patient’s limbs. Moreover, sensory perception is also profoundly damaged, with negative repercussions on motor control and the ability to interact with the surrounding environment [3]. In the last decades, with the aid of micromachining technologies, multiple solutions have been developed to restore lost sensorimotor functions and to improve patients’ lives. In this context, implantable neural interfaces are devices able to record and modulate axon potentials by establishing a bidirectional electrical connection at the interface between the tissue and the device [4]. Among them, intraneural electrodes enable lower charge injection and higher signal-to-noise ratio for stimulation and recording, respectively, in comparison to extraneural electrodes [5]. These effects are due to the possibility of directly interfacing with a single nerve fascicle, thus allowing excellent recording/stimulation selectivity. These electrodes have recently been used in bidirectional closed-loop neuromodulation applications to restore tactile perception, improve walking speed, and reduce phantom-limb pain in patients with mechatronic prosthesis [6,7].

Several designs, materials, and fabrication procedures have been presented to facilitate the development of personalized neuroprosthesis [8]. Implantable flexible neural interfaces (IfNIs) have recently gained much attention in the framework of neuromodulation devices, due to their reduced rigidity in comparison to stiffer silicon-based devices [9,10,11]. In this regard, the use of polyimide (PI) as an insulating substrate has become extremely popular due to its excellent dielectric properties, low water uptake, mechanical strength, and resistance to harsh environments. Interesting examples of PI-based devices are TIME and LIFE electrodes, respectively transversally and longitudinally inserted IfNIs [12,13,14].

Unfortunately, notwithstanding the remarkable neural signal modulation performance on animal and human acute experiments, the long-term application of such devices remains the main bottleneck of implantable device technology [15]. Although tissue penetration is required to interface with nerve fascicles to obtain a high level of selectivity, this procedure inevitably damages nerve integrity, thus triggering a defense mechanism called foreign body reaction (FBR). FBR is a cascade of events characterized by a first phase of blood plasma protein (fibronectin, albumin, fibrinogen, and complement factors) absorption onto the surface of the device [16]. This process leads to an inflammatory phase unleashed by monocytes and neutrophils, followed by a fibrotic phase triggered by fibroblast recruitment that modulates the coagulation cascade with subsequent formation of a fibrotic capsule that surrounds the surface of the implant. This process is sustained by monocyte differentiation to macrophages and pro-inflammatory cytokine (TNF-α, IL-1, IL-6, IL-8) release at the site of the implant. The formation of a connective tissue layer around the device implies a drop in its electronic performance, with a consequent increase in the stimulation threshold and decrease in the quality of the recorded signal that limit the long-term usage of IfNIs [17,18]. Moreover, due to the different physiochemical properties and the substantial mechanical mismatch between PI (E_PI_ = 2.5 GPa) and the nerve (E_nerve_ = 500 kPa), FBR effects are chronically sustained with consequent tissue damage and the need for a second invasive surgery to remove the implant after few months.

In the literature, several examples and technologies have been reported to improve IfNI biocompatibility [19]. In this context, the study and development of a proper coating to enhance the long-term biocompatibility of IfNIs represent a primary objective to envision chronic stable implants. The aim of a coating material is to interpose a *buffer layer* between the tissue and the surface of the device to reduce the mechanical mismatch, to improve the tissue/electrode interface, to functionalize the device’s surface with biomolecules or drugs to aid neural cell attachment, to reduce scar tissue formation, and to attenuate the FBR effects over time [20]. In addition, it is possible to modify the surface of PI with proteins or peptides to facilitate Schwann cell and neuron adhesion [21,22,23]. Lots of research has also been conducted on the hydrogel surface coating of IfNIs. Hydrogels are 3D structures made by synthetic, natural, conductive, or bioactive materials that mimic the physiochemical properties of the extracellular matrix (ECM), whose aim is to improve device tissue integration, thus enhancing long-term biocompatibility [24]. In this regard, Shen and colleagues reported a remarkable example of a Matrigel^®^/collagen ECM-like coating for intracortical electrodes to enhance chronic integration of a device implanted in the brain [25]. Furthermore, a drug-loaded hydrogel was also reported with the fabrication of a poly(ethylene glycol) (PEG) hydrogel containing poly(lactic-co-glycolic) acid (PLGA) microspheres loaded with an anti-inflammatory drug, to reduce long-term FBR effects [26]. Another interesting example illustrating a polyacrylamide and poly(3,4-ethylenedioxythiophene) polystyrene sulfonate (PEDOT:PSS) conductive hydrogel to improve the biocompatibility and electrochemical performance of an elastomeric IfNIs was recently described [27]. Hydrogel IfNIs coatings have also successfully been used as cell-repellent materials, exploiting the anti-fouling properties of highly hydrophilic surfaces to reduce scar tissue formation on the electrode surface [28,29,30].

The aim of this work was to present a nanostructured coating made from natural polysaccharides to improve the biocompatibility of PI-based IfNIs. A layer-by-layer (LbL) technique was used to fabricate this coating upon PI surface modification by oxygen plasma. LbL assembly consists of the sequential electrostatic adsorption of polyelectrolytes, in order to fabricate multilayered coatings [31,32]. It was chosen in this framework due to its low-cost implementation and excellent versatility to tune structural and surface properties, such as thickness, elastic modulus, wettability, swelling, and surface roughness, and thanks to the possibility to incorporate biomolecules and nanostructures within the multilayer assembly [33,34,35,36,37,38,39]. Polysaccharides such as sodium alginate and chitosan were selected to coat the PI surface by means of LbL assembly due to their remarkable biocompatibility and similar physiochemical properties to tissue ECM. Sodium alginate derived from *brown algae* is a widely used example of a polyanionic polysaccharide for cell encapsulation and drug delivery applications [40]. Chitosan, derived from the deacetylation of chitin, is extracted from crustacean shells and has been widely used in neural engineering as a promising natural material for peripheral nerve regeneration, due to its good cytocompatibility with neurons and Schwann cells [41,42,43,44]. Furthermore, polysaccharides have already been reported to possess anti-fouling properties thanks to their high hydrophilicity, which decreases surface protein adsorption [45,46,47,48]. Morphological and physiochemical analysis of the nanostructured coating was carried out to characterize the structural parameters of the coating, such as thickness, wettability, and surface roughness. Two different LbL deposition methods (dip coating and spin coating) were used and compared to investigate surface property modifications with the deposition technique. To the best of our knowledge, this is the first example showing the use of the LbL technique to fabricate a biocompatible nanostructured coating for PI-based neural interfaces. This coating strategy could represent a very versatile, low-cost, and easy-to-implement method to improve the long-term application of IfNIs.

## 2. Materials and Methods

### 2.1. Materials

Polyimide resin PI 2610 was purchased from DuPont MicroSystems GmbH. Sodium alginate (MW = 80–100 kDa), chitosan (MW = 310–375 kDa, deacetylation degree > 75%) and all the other chemicals were purchased from Sigma Aldrich.

Silicon wafers (400 μm thick, p-type, boron doped, ⟨100⟩, Si-Mat Silicon Materials, Kaufering, Germany), used as substrates for PI film deposition, were cut (3 cm × 3 cm) and dipped in an acetone/isopropanol solution for 15 min, washed with deionized (DI) water (18 MΩ cm) and dried with filtered compressed air to remove dust and impurities. All the fabrication steps regarding PI deposition, plasma treatment, and subsequent LbL coating were performed in a class 10,000 clean room to avoid contamination.

**Scheme 1:**
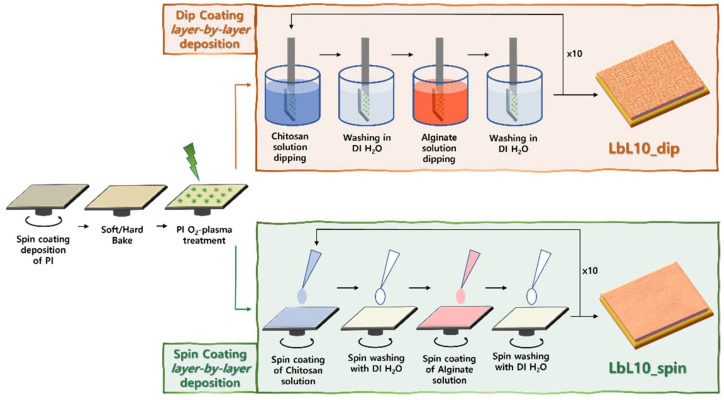
PI substrate and polysaccharide LbL coating production. Fabrication steps of PI deposition and curing onto silicon substrates, oxygen plasma treatment, and subsequent LbL assembly by dip coating and by spin coating.

All PI and LbL coating fabrication steps are illustrated in Scheme 1.

### 2.2. Methods

#### 2.2.1. Polyimide Film Deposition and Plasma Treatment

PI was spin-coated onto a silicon substrate using a two-step program (500 rpm, 10 s and 4500 rpm, 30 s). After the spinning procedure, the samples were treated at 65 °C and 130 °C for 3 min on a hot plate (soft-bake) and subsequently cured at 350 °C in a nitrogen atmosphere for 60 min (hard-bake) to obtain a homogeneous thin film. After spinning deposition, PI substrates were treated with oxygen plasma (Colibrì, Gambetti, Binasco, Italy) at 40 W for 120 s at 0.6 mbar to increase surface hydrophilicity and to allow better anchorage of the polysaccharides. Two different oxygen gas percentages of 20% (PI_plasma 20%) and of 50% (PI_plasma 50%) were used during the treatment to assess differences in PI surface roughness and wettability prior to proceeding with LbL polysaccharide deposition.

#### 2.2.2. LbL Thin Film Deposition by Dip Coating

After plasma treatment, PI substrates were immediately coated with alternated dipping in polysaccharide solutions to fabricate LbL-coated PI. We refer to PI samples coated with dip coating LbL deposition as “LbL_nbilayers_dip”. Briefly, PI substrate was dipped into a chitosan solution (10 mg/mL, 1% *v*/*v* CH_3_COOH in DI water), then rinsed by immersion in DI water and then dipped in sodium alginate solution (10 mg/mL in DI water), followed by another rinsing procedure in DI water. All the dipping steps were conducted for 15 min at room temperature. This procedure was used to deposit one chitosan/alginate bilayer onto the surface of PI and was repeated for each the 10 bilayers sequentially adsorbed onto the substrate surface. 

#### 2.2.3. LbL Thin Film Deposition by Spin Coating

Spin-assisted LbL deposition was also used, in comparison with dip coating, to verify the modification of the surface properties with the coating technique. We refer to PI samples coated with spin-assisted LbL deposition as “LbL_nbilayers_spin”. Briefly, after plasma treatment, 500 µL of chitosan solution (2 mg/mL, 1% *v*/*v* CH_3_COOH in DI water) was deposited onto the PI substrate and spin-coated at 4500 rpm for 35 s. After this procedure, the wafer was rinsed twice with a drop of 500 µL of DI water spin-coated with the same parameters. Then a drop of 500 µL of sodium alginate solution (2 mg/mL in DI water) was deposited and spin-coated at 4500 rpm for 35 s, followed by the same rinsing method with DI water mentioned before. As for dip coating, this procedure was used to deposit one chitosan/alginate bilayer onto the surface of PI and was repeated for each of the 10 bilayers sequentially adsorbed onto the substrate surface. 

#### 2.2.4. PI and LbL Thin Film Characterization

Substrate thickness was measured with a P6 surface profiler (KLA-Tencor, Milpitas CA, USA). In particular, to measure PI samples, a little scratch onto their surface was made using metallic tweezers and the height profile across the scratch was recorded. For LbL thickness measurements, the same scratch was made using a sharp plastic tip, in order to delaminate the LbL thin film from the surface of the PI without damaging the underlying PI substrate. Sample thickness was measured at 5 arbitrary points in each sample to calculate mean and standard deviation (SD). For LbL-coated samples, thickness was measured after deposition of 4, 7, and 10 bilayers.

Substrate wettability was analyzed using the sessile drop method by means of an Attension Theta optical tensiometer (Biolin Scientific). A tiny droplet of 2 μL of DI water was deposited onto the surface of the sample and the spreading of the droplet was imaged at 14 frames/sec for a total range of 15 sec. The angles were measured at 5 different and arbitrary points for each sample to calculate mean and SD. For LbL-coated samples, contact angle (CA) was measured after deposition of 4, 7, and 10 bilayers.

Atomic force microscopy (AFM) was used to investigate surface topography with an Innova SPM (Bruker, Billerica, CA, USA) operating in tapping mode using gold-coated n-type silicon probes (NSC01, f_0_ = 87 − 230 kHz, k = 1.45 − 15.1 Nm^−1^, NT-MDT, Moscow, Russia). All scans were performed at room temperature, on samples supported on the silicon substrates, scanning 5 μm × 5 μm areas at a scan rate of 0.5 Hz on 5 different and arbitrary zones to calculate mean and SD. All AFM data were elaborated using the Gwyddion SPM analysis tool (http://gwyddion.net (accessed on 14 January 2022)). Samples’ average surface roughness (*R_a_*) was measured after deposition of 4, 7, and 10 bilayers. *R_a_* was calculated using the following equation:

(1) $$R_{a}=\frac{1}{L}\;\int_{0}^{L}\left|Z_{i}\right|dx$$

where *L* is the sampling length and *Z_i_* is the current Z value.

Infrared spectra of samples were taken in transmittance mode (T%) using an IRPrestige-21 IRAfinity-1 FTIR-8400S (Shimadzu, Japan). Measurements were taken with a spectral range of 500–4000 cm^−1^ by accumulation of 16 scans and a resolution of 4 cm^−1^. Omni Spectra software was used to analyze the IR spectra of LbL-coated PI and compare these with the untreated PI, chitosan, and alginate peak bands, which were used as reference controls. For each specimen 3 measurements were performed, each one in a different and arbitrary position within the total area. Results of FTIR characterization are reported in the Supplementary Materials Section.

#### 2.2.5. Statistical Analysis

Data were statistically analyzed using the commercial software GraphPad Prism 8 (San Diego, CA, USA). One-Way ANOVA (Tukey’s multiple comparison test) or two-tailed unpaired *t*-test were used to evaluate the statistical significance between the samples in each group. All data are reported as mean ± SD. In all experiments, statistical significance refers to results where *p* < 0.05 was obtained. In particular, statistical significance thresholds were set as follows: * = *p* < 0.05; ** = *p* < 0.01; *** = *p* < 0.0005, and **** = *p* < 0.0001.

## 3. Results

### 3.1. Spin Coating Deposition and Plasma Treatment of PI Substrates

Spin coating of PI and further LbL coating were performed onto square-shaped silicon substrates to facilitate the handling, fabrication, and characterization procedures. Soft- and hard-bake steps on spin-coated PI led to the fabrication of a highly homogeneous, tight, and smooth surface on the silicon substrate.

**Figure 1:**
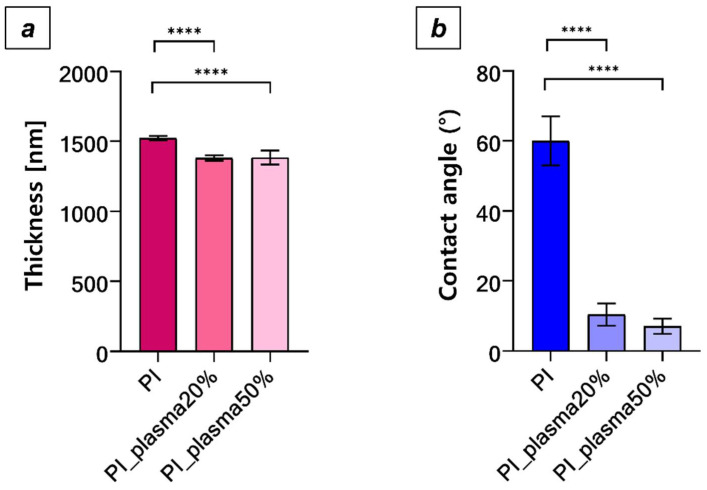
PI substrate characterization. (**a**) Thickness measurement of spin-coated PI samples after hard-bake procedure (PI) and after plasma treatment using different oxygen gas percentages (PI_plasma20% and PI_plasma50%). (**b**) CA measurements of untreated PI and after plasma treatment.

Figure 1a shows the average thickness of PI substrates after curing and plasma treatment. Oxygen plasma significantly reduced the average PI thickness due to the erosive attack of free oxygen species on the polymeric surface for both the oxygen percentages used. The reported average thicknesses of PI substrates were 1523 ± 15.3 nm, 1381 ± 19.86 nm, and 1384 ± 49.89 nm for untreated PI, PI_plasma20%, and PI_plasma50%, respectively.

**Figure 2:**
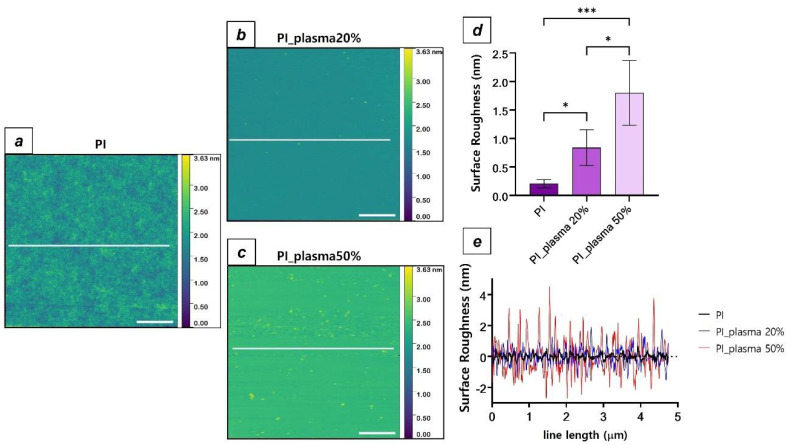
Surface roughness analysis of PI. AFM scan of untreated PI (**a**), PI_plasma20% (**b**), and PI_plasma50% (**c**). (**d**) Average surface roughness calculated for untreated and plasma-treated PI. (**e**) Plot of surface roughness profile of PI substrates relative to the white line drawn in each AFM scan. Scale bars are 1 µm.

Air plasma treatment induces PI depolymerization and formation of low molecular weight oxidized material (LMWOM) [49,50]. Figure 1b shows CA data for PI substrates upon plasma treatment and a marked increase in surface hydrophilicity was reported after oxygen plasma exposure. In particular, untreated PI CA of 60.01 ± 7.02° was measured and a statistically different wettability increase was described after 20% oxygen plasma (CA = 10.37 ± 3.2°, *p* < 0.0001) and after 50% oxygen plasma (CA = 7.02 ± 2.19°, *p* < 0.0001). This effect was due to the increase in oxygen content of the treated surface, with the formation of hydroperoxides, carbonyls, carboxylic acids, peracids, etc., which remarkably increase surface wettability with respect to untreated PI [51]. Statistically significant differences were not reported by increasing the oxygen content from 20% to 50% both for PI substrate thickness and CA. In addition to the thickness reduction and wettability increase of PI upon plasma treatment, significant modification of the surface roughness was reported thanks to AFM analysis (Figure 2).

AFM analysis reports modification of PI surface topography with nanometric features after plasma treatment with respect to untreated PI, due to the erosive behavior of oxygen plasma (Figure 2d,e). In detail, a statistically significant roughness increase was described either after plasma treatment with 20% oxygen (*R_a_* PI = 0.2 ± 0.07 nm; *R_a_* PI_plasma20% = 0.8 ± 0.31 nm, *p* < 0.05) or after treatment with 50% (*R_a_* PI_plasma50% = 1.8 ± 0.56 nm, *p* < 0.0005). In this case, statistical significance was also reported within the two treatments (*p* < 0.05). 

In summary, plasma treatment of spin-coated PI substrates generates a thickness reduction and a consistent increase in surface hydrophilicity and roughness due to LMWOM generation and to the formation of charged chemical groups. Treatment with a 50% oxygen percentage was selected for further LbL deposition due to its significant roughness increase with respect to the treatment with 20% oxygen. The reason for this choice lies in the fact that a greater surface roughness increases the contact area between the PI and the polymer solutions used for the coating fabrication and could therefore lead to better surface adhesion of the polysaccharide multilayer.

### 3.2. LbL Polysaccharide Deposition by Dip Coating

After plasma treatment, PI substrates were alternatively dipped, with intermediate washing steps, in the polyelectrolyte solutions for 15 min in order to create the LbL assembly by dip coating. Polymer adsorption onto plasma-treated PI was possible thanks to electrostatic interactions between charged species on PI surfaces and between oppositely charged polysaccharide chains [39]. 

**Figure 3:**
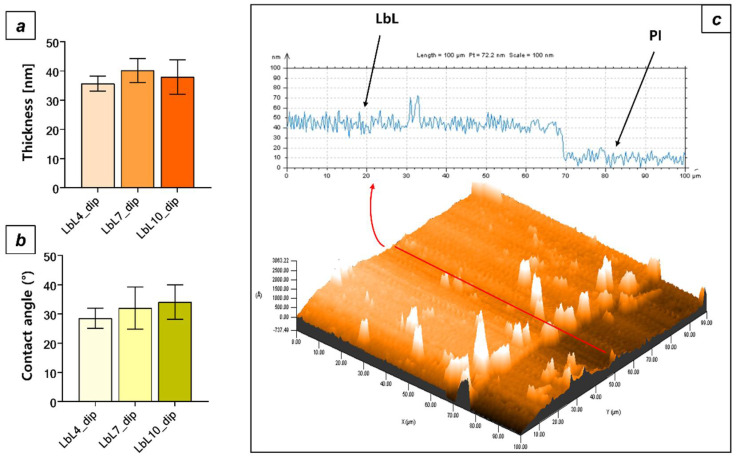
LbL assembly by dip coating. Thickness data (**a**) and CA values (**b**) of LbL assembly onto PI surface after the deposition of 4 (LbL4_dip), 7 (LbL7_dip), and 10 (LbL10_dip) polysaccharide bilayers. (**c**) 3D reconstruction of a 100 µm × 100 µm surface profilometry referred to a LbL10_dip sample and plot of surface roughness profile relative to the red line across the scratch showing the presence of an LbL multilayer coating on the surface of the PI.

Surface profilometry confirmed the presence of a multilayer assembly on the surface of the PI (Figure 3c) with an average thickness of around 40 nm after the deposition of 10 bilayers. However, no statistical differences were reported after subsequent deposition of 4 and higher numbers of bilayers (thickness: LbL4_dip = 37.88 ± 2.54 nm; LbL7_dip = 40.15 ± 4.08 nm; LbL10_dip = 35.68 ± 5.90 nm), whose average heights did not follow an increasing trend with the number of dipping cycles (Figure 1a). CA analysis reports similar evidence regarding surface wettability after LbL dip-coating deposition (Figure 3b). No statistical difference was reported after the deposition of an increasing number of bilayers onto the PI surface (CA: LbL4_dip = 28.52 ± 3.46°; LbL7_dip = 32 ± 7.23°; LbL10_dip = 34 ± 5.90°).

**Figure 4:**
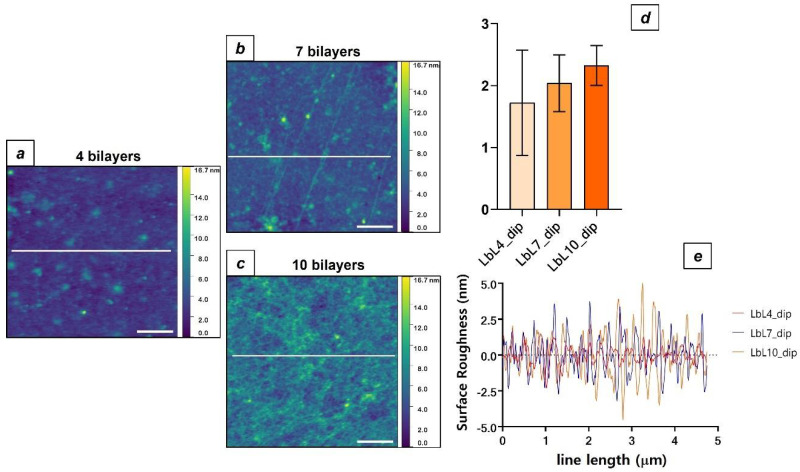
AFM topographical analysis of LbL assembly by dip coating. AFM scan of LbL surface after the deposition of 4 (**a**), 7 (**b**), and 10 (**c**) bilayers. (**d**) Average surface roughness of LbL multilayers with increasing number of bilayers deposited onto PI surface. (**e**) Plot of surface roughness profile of dip-coated LbL substrates relative to the white line drawn in each AFM scan. Scale bars are 1 µm.

AFM analysis after dip-coating LbL deposition showed no statistical differences in surface roughness value after subsequent deposition of a higher number of bilayers, even though significant topographical differences were evidenced as the number of bilayers deposited onto the PI surface increased (Figure 4).

These results evidence the presence of a polysaccharide coating on the surface of the PI assembled by the dip-coating LbL technique, with nanometric thickness and roughness and a hydrophilic surface.

### 3.3. LbL Polysaccharide Deposition by Spin Coating

The spin-assisted LbL method [52] was also used to assemble the polysaccharide coating onto the plasma-treated PI surface, to assess whether there were differences in coating physiochemical and topographical properties with respect to dip-coating LbL deposition.

**Figure 5:**
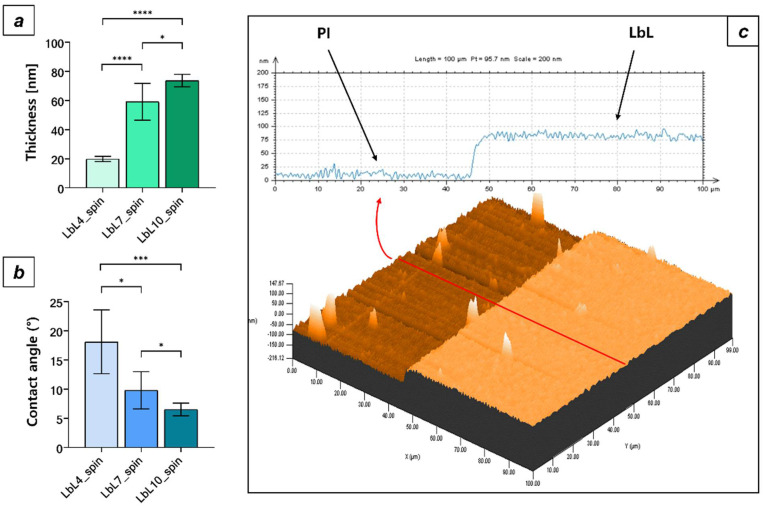
LbL assembly by spin coating. Thickness data (**a**) and CA values (**b**) of LbL assembly onto PI surface after the deposition of 4 (LbL4_spin), 7 (LbL7_spin), and 10 (LbL10_spin) polysaccharide bilayers. (**c**) 3D reconstruction of a 100 µm × 100 µm surface profilometry referred to a LbL10_spin sample and plot of surface roughness profile relative to the red line across the scratch showing the presence of an LbL multilayer coating on the surface of the PI.

The thickness analysis reports the presence of a polysaccharide multilayer thin film on the surface of the PI after alternate spin-coating deposition of chitosan and sodium alginate solutions. LbL assembly by spin coating shows a smoother and more homogeneous profile (Figure 5c) with respect to the same multilayer coating deposited by dip coating (Figure 3 and Figure 4). This effect is due to the huge shear stresses during the spin-coating process, which contribute to aligning the polymer chains in the direction of the centrifugal force [53]. The presence of a multilayer assembly on the PI surface is also confirmed by FT-IR analysis (see Figure S1).

Unlike dip-coating deposition, spin-coated LbL multilayers reported a statistically significant increasing trend of the average thickness as the number of pairs of polyelectrolytes that are deposited on the surface of the PI increased. Average thickness values of 19.83 ± 1.91 nm, 59.11 ± 13.68 nm, and of 73.66 ± 4.30 nm were reported for LbL4_spin, LbL7_spin, and LbL10_spin samples, respectively (Figure 5a). Conversely, a decrease in average CA values was reported as the numbers of spin-coated bilayers increased (Figure 5b), consistent with previous reports in the literature regarding polysaccharide thin film coating [47,54]. A CA decrease as the film thickness increases can also be explained with the progressively lower influence of the PI surface on the overall wettability, as the thickness of the hydrophilic polysaccharide layer increases with multiple spin-coating deposition steps. 

**Figure 6:**
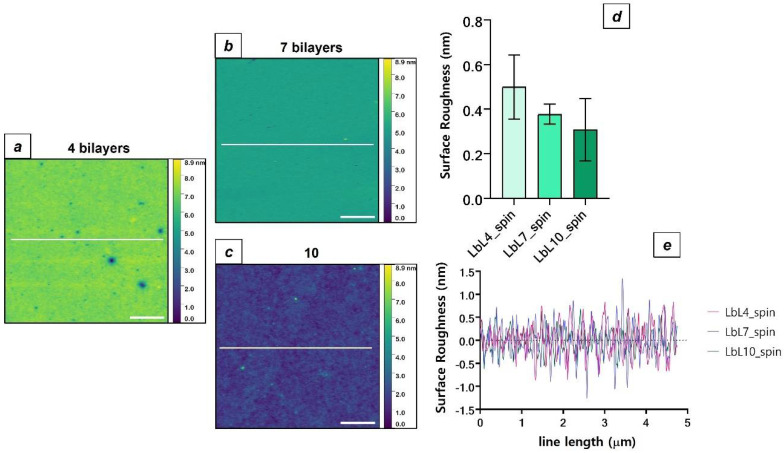
AFM topographical analysis of LbL assembly by spin coating. AFM scan of LbL surface after the deposition of 4 (**a**), 7 (**b**), and 10 (**c**) bilayers. (**d**) Average surface roughness of LbL multilayers with increasing number of bilayers deposited onto the PI surface. (**e**) Plot of surface roughness profile of spin-coated LbL substrates relative to the white line drawn in each AFM scan. Scale bars are 1 µm.

AFM analysis also reveals in this case the presence of a nanometric surface roughness for the LbL assembly deposited by spin coating onto the surface of the PI. The topography of spin-coated LbL samples appears to be very smooth due to the huge shear stresses that characterize the spin-assisted LbL method (Figure 6a–c), with average roughness values less than 1 nm for each number of bilayers sequentially deposited (*R_a_* LbL4_spin = 0.5 ± 0.14 nm; *R_a_* LbL7_spin = 0.37 ± 0.04 nm; *R_a_* LbL10_spin = 0.3 ± 0.14). However, no statistical differences were reported between the average roughness values of the spin-coated LbL coating by increasing the number of bilayers deposited on the PI surface (Figure 6d).

Results for the spin-coated LbL assembly show the presence of very smooth and highly hydrophilic (CA < 10° for LbL10_spin) polysaccharide multilayers on the PI surface, with an extremely low surface roughness (*R_a_* < 1 nm for each number of bilayers deposited). Profilometry and CA measurements demonstrated the robustness of the implemented spin-coated recipe with respect to the dip-coating one, since statistically significant differences in average thickness and CA were reported as the number of deposited bilayers increased (*p* < 0.05 for increasing number of bilayers deposited for both thickness and CA values reported).

### 3.4. Comparison between PI Surface Properties and the Two LbL Deposition Techniques

**Figure 7:**
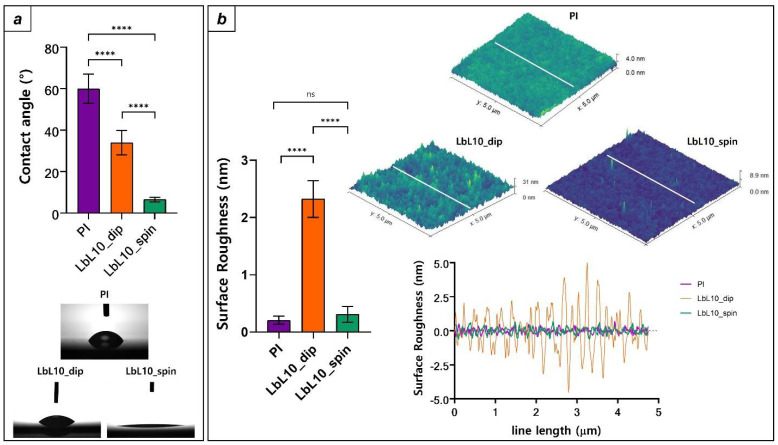
Physiochemical properties of the two LbL assembly methods compared with the PI surface. (**a**) CA values of the two LbL techniques compared with PI. At the bottom of the box are shown representative images of water-drop spreading for each sample typology, evidencing differences in surface wettability. (**b**) Summary of AFM analysis reporting variations in average surface roughness for each sample in the histogram and 3D reconstruction of 5 µm × 5 µm surface topography of PI, LbL10_dip, and LbL10_spin. At the bottom of the box plot of surface roughness, the profile relative to the white line drawn in each AFM scan is shown.

Both the LbL assembly methods used in this work reported successful polysaccharide thin film anchorage onto the PI surface (Figure 3 and Figure 5). However, substantial differences can be evidenced when comparing the physiochemical properties of untreated PI and LbL surfaces after the deposition of 10 bilayers (Figure 7).

As it is possible to notice from Figure 7a, LbL polysaccharide coating of PI led to a marked increase in surface hydrophilicity (*p* < 0.0001) for both the deposition methods tested. In detail, LbL coating was able to reduce CA values to 34.03 ± 5.9° and to 6.5 ± 1.1° for dip-coated and spin-coated samples, respectively, demonstrating a remarkable increase in surface wettability with respect to untreated PI (CA_untreated PI_ = 60.01 ± 7.02°). This behavior was also illustrated by water-drop spreading after LbL deposition (Figure 7a) and is due to the presence of polar and highly hydrophilic groups (hydroxyl, carboxyl, and amino groups) in the polysaccharide chains. Moreover, LbL deposition led to consistent topographical changes with respect to the PI surface (Figure 7b). AFM analysis reported enhanced average surface roughness upon LbL dip-coating deposition (*R_a_* PI = 0.3 ± 0.07 nm, *R_a_* LbL10_dip = 2.32 ± 0.32, *p* < 0.0001). This effect was due to the high polymer chain interpenetration typical of LbL dip-coating assembly, which led to a roughness increase with respect to untreated PI [55,56]. Conversely, untreated PI and spin-coated LbL display very similar roughness values (*R_a_* PI = 0.2 ± 0.07 nm, *R_a_* LbL10_spin = 0.3 ± 0.13 nm) in line with previous reports regarding PI and spin-assisted polysaccharide LbL [21,35]. Furthermore, marked discrepancies in topography and CA values were also reported between different LbL deposition methods. In this regard, a higher wettability and smoother surface were evidenced for spin-coated substrates with respect to dip-coated ones (*p* < 0.0001), consistent with previous literature reports [53,57,58]. This effect is due to the different environmental conditions of the two LbL assembly methods, which remarkably affect surface properties: huge shear stresses during the spin-coating procedure create a much smoother surface with respect to dip coating, with enhanced surface wettability due to lower surface roughness [59,60]. 

In summary, LbL assembly allowed the successful deposition of a nanostructured thin polysaccharide coating on plasma-treated PI with both the LbL techniques chosen (Table 1). Remarkably, increased surface hydrophilicity and a very smooth and homogeneous surface were demonstrated for spin-coated LbL, whereas a less hydrophilic and more irregular surface was reported for dip-coated LbL. Finally, both the LbL coating methods allowed significant enhancement of surface wettability with respect to the PI surface thanks to the polysaccharide chemical structure. Interestingly, spin-coated polysaccharide LbL surfaces display marked increases in hydrophilicity with unaltered roughness with respect to untreated PI.

## 4. Discussion

This study described a novel method to coat PI-based IfNIs employing a nanostructured polysaccharide coating with the aim to enhance their biocompatibility and long-term usage. Two marine polysaccharides, chitosan and sodium alginate, were selected due to their interesting physiochemical properties, such as high hydrophilicity and similar mechanical properties to ECM components [61,62,63,64]. Moreover, these polysaccharides have already been reported to possess remarkable cytocompatibility with neural cells such as primary hippocampal neurons [65], cortical neurons [66] and Schwann cells [67,68].

Both the studied LbL techniques allowed the deposition of a nanostructured thin film coating onto the surface of PI (thickness LbL10_dip = 35.68 ± 5.90 nm, thickness LbL10_spin = 73.66 ± 4.30 nm), but spin-coating assembly guaranteed higher control over the fabrication parameters, enabling the possibility to tune some important characteristics of the polysaccharide assembly, such as thickness, surface roughness, and wettability (Figure 5 and Figure 6). Conversely, the dip-coating technique used in this work, although reported to successfully coat the PI surface with a nanostructured thin film assembly with increased hydrophilicity with respect to untreated PI, did not allow us to control the morphological characteristics of the coating with the manufacturing parameters. However, this technique can be improved by implementing better control on the ionization of the polysaccharide chains, by tuning the ionic strength and the pH of the polyelectrolyte solutions [69,70,71]. 

Overall, the studied LbL coating strategy allowed PI surface modification with polysaccharide thin films characterized by extremely high surface hydrophilicity (CA < 10° for the spin-coated LbL coating), nanostructured thickness (<100 nm for both the techniques), and a remarkably smooth surface (*R_a_* < 1 nm for spin coated LbL). All these documented features allow the envisioning of this coating strategy to provide a cell repellent and anti-fouling barrier for IfNIs, which would reduce plasma blood protein adsorption upon implantation and decrease FBR effects over time. Protein adsorption reduction is a key feature for anti-fouling materials as this occurrence significantly reduces macrophages and fibroblast incursion [48]. Several reports in the literature describe the use of PEG as an excellent anti-fouling coating for devices and nanostructures thanks to the formation of a thick hydration layer that strongly reduces protein adsorption [72,73,74,75]. Polysaccharide coatings have also been reported to exhibit good anti-fouling properties, reducing cell and bacterial adhesion with the same mechanism [59]. As an example, chitosan/pectin multilayers [60], chitosan/carboxymethyl cellulose [47] and chondroitin sulfate [46] polysaccharide thin film coatings have been reported to exhibit very low protein adsorption upon immersion in physiological conditions when compared with untreated surfaces. Protein adsorption kinetics on surfaces is a complex mechanism that involves intermolecular forces that generate the three-dimensional structure of the protein and its interaction with the substrate at the liquid–solid interface [76,77,78]. Surface hydrophilicity and hydrogen-bond-forming species are prerequisite for anti-fouling properties, as hydrophobic interactions may cause irreversible protein adsorption [72]. As an example, PI and polydimethylsiloxane (PDMS) surfaces have been reported to absorb high quantities of blood plasma proteins due to their relative hydrophobic behavior [79,80]. Physical methods, such as oxygen plasma, have been employed to increase surface wettability in order to reduce protein adsorption, but their efficacy is limited, since the original wettability is restored after a certain period of time due to a phenomenon called “hydrophobic recovery” [81,82]. 

Therefore, coating IfNIs surfaces with a stable nanostructured hydrophilic layer could be a promising way to enhance their long-term performance by exploiting anti-fouling properties, since FBR effects upon IfNIs implantation have been reported to worsen their electrical performance over time [9]. In the literature, the PI surface has already been modified with anti-fouling materials, employing zwitterionic hydrogel [30]. The coating demonstrated reduced macrophage adhesion in vitro and the morphological characterization reported a coating thickness in the micrometer range that increased by one order of magnitude upon consistent swelling due to the marked hydrophilic behavior of zwitterionic polymers. Although the presence of a highly wettable layer on the PI surface is recommended to exploit its anti-fouling properties, the use of thick and very soft coating poses several limitations over the stability and the electronic performance of the implanted device [83]. Macroscopic hydrogel coatings are reported to consistently swell in a water environment, increasing electrical impedance over time and causing progressive detachment from the electrode surface during the implantation procedure [84]. This process could reduce the recording capability of the device, since the huge coating swelling would increase the distance between the tissue and the active conductive sites [25,85,86,87]. In this view, LbL coating would provide a smart way to coat a neural interface with a nanostructured and highly hydrophilic multilayer with reduced thickness with respect to macroscopic coating. Previous reports describing polysaccharide LbL assembly showed moderate swelling (Thickness < 100 nm) in a water environment due to the nanostructured feature of spin-assisted LbL multilayers [39]. Moreover, LbL techniques offer several advantages over other described coating strategies, such as the possibility to be chemically functionalized with peptides to enhance tissue integration and to incorporate drugs and nanostructures within the polymeric matrix, thus providing multiple functionalities to the nanostructured coating [87].

In summary, the described LbL coating strategy was demonstrated to successfully functionalize the PI electrode surface with a nanostructured, highly hydrophilic, and very smooth surface that could enhance the long-term biocompatibility of IfNIs. Furthermore, thanks to the ease of fabrication inherent in the LbL method, this technique could be easily implemented in the current IfNIs manufacturing processes based on micromachining technology, allowing quick translational applicability. Further in vivo tests will be needed to characterize its behavior in chronic animal experiments of neuromodulation.

## 5. Conclusions

This work described a novel method to fabricate a biocompatible coating for PI-based IfNIs. The LbL assembly technique was chosen to fabricate the coating due to its well-documented versatility and ease of manufacturing. Two different LbL methods (dip coating and spin coating LbL assembly) were implemented and compared to assess the physiochemical and topographical properties of the coating with respect to the PI surface properties. The results of the study show the possibility to provide a nanostructured, highly hydrophilic, and ultra-smooth polysaccharide multilayer over the surface of the PI, with increased wettability and comparable surface smoothness with respect to untreated PI. Moreover, spin-coating deposition offered more robustness during the fabrication procedure, allowing fine-tuning of the coating physiochemical properties by increasing the number of bilayers deposited. Future in vivo experiments will be needed to assess the functionality of the proposed coating strategy.

## Figures and Tables

**Table 1:** Summary of physiochemical properties of untreated and LbL-coated PI.

Sample Type	Contact Angle (°)	*R_a_* (nm)
PI	60.01 ± 7.02	0.*2* ± 0.*07*
LbL10_dip	34.03 ± 5.9	*2.32* ± 0.*32*
LbL10_spin	6.5 ± 1.1	0.3 ± 0.*13*

## Data Availability

All supporting data is included in the paper.

## Supplementary Materials

The following supporting information can be downloaded at https://www.mdpi.com/article/10.3390/mi13050692/s1, Figure S1: FT-IR analysis. Transmission spectra of PI and LbL coated samples and of the two polysaccharides used for LbL assembly (in the inset). The red arrow and ellipse evidence the characteristics polysaccharide peaks, while black arrows are relative to PI peaks.
