# Supplementary material for: Polysaccharide Layer-by-Layer Coating for Polyimide-Based Neural Interfaces
Source: Micromachines (Basel). 2022 Apr 28;13(5):692. doi: 10.3390/mi13050692 (PMC9146946; doi:10.3390/mi13050692)
Supplement: Supplementary file 1 [file micromachines-13-00692-s001.zip › micromachines-1670339-supplementary.pdf]

micromachines

MDPI

Supplementary Material

FT-IR analysis

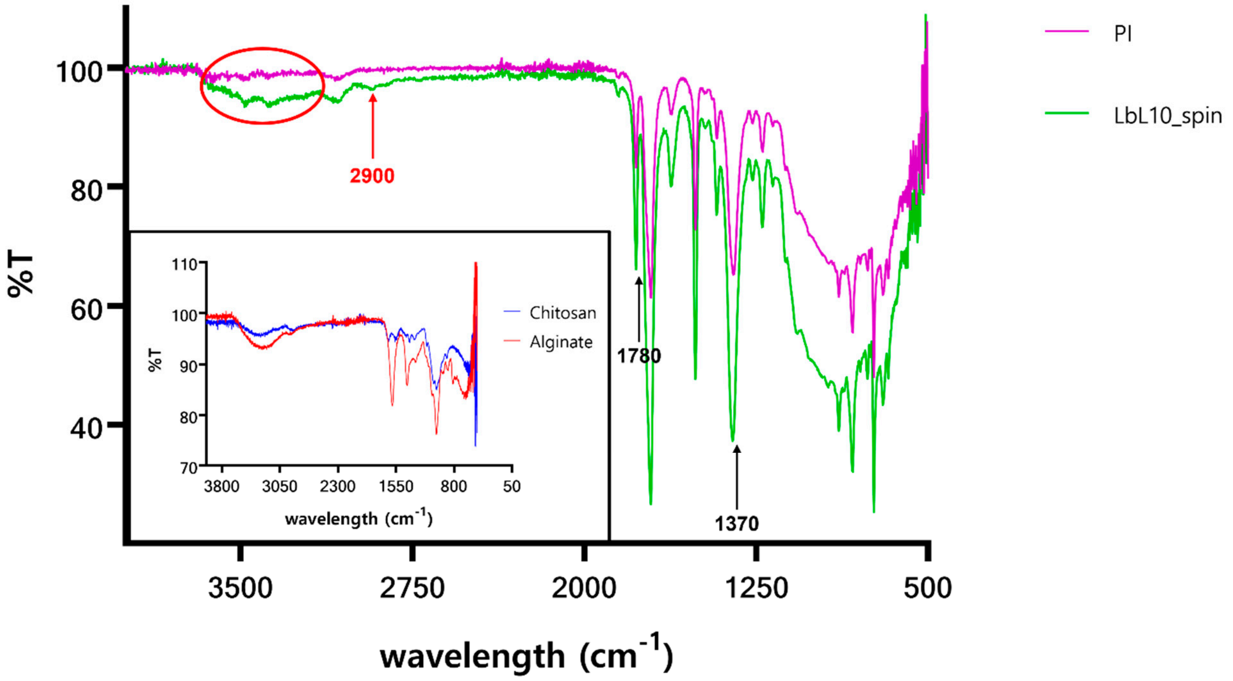

Figure S1. FT-IR analysis. Transmission spectra of PI and LbL coated samples and of the two polysaccharides used for LbL assembly (in the inset). The red arrow and ellipse evidence the characteristics polysaccharide peaks, while black arrows are relative to PI peaks.

As it is possible to notice from the comparison of the FT-IR spectrum of untreated PI with the multilayer deposited by spin-coating in Figure S1, the presence of some significant peaks can be noted. In the first place, it is possible to appreciate a peak in correspondence of 2900 cm^{-1}, absent in the spectrum of the polyimide alone and present instead in the spectra of pure alginate and chitosan, relating to the stretching of the C-H bond, typical of the polysaccharides molecules. Furthermore, it can be noted the presence of a band between the wavelengths of 3250-3700 cm^{-1} in the LbL10_spin spectrum not present in the untreated PI spectrum. This evidence is due to the presence of hydroxyl groups (-OH) the polysaccharides chains and whose stretching of the -OH bond generates a decrease in transmittance around the aforementioned band. From the spectra it is also possible to note the characteristic peaks of the polyimide: in particular the one at 1370 cm^{-1} is related to the contribution of the C-N group (imide) and another one at the wavelength of 1780 cm^{-1} related to the contribution of the carbonyl group (C=O). These evidences allow to confirm the presence of the polysaccharide coating onto the surface of PI.
